# Neurons of self-defence: neuronal innervation of the exocrine defence glands in stick insects

**DOI:** 10.1186/s12983-015-0122-0

**Published:** 2015-10-24

**Authors:** Konrad Stolz, Christoph-Rüdiger von Bredow, Yvette M. von Bredow, Reinhard Lakes-Harlan, Tina E. Trenczek, Johannes Strauß

**Affiliations:** Institute for General and Applied Zoology, Justus-Liebig-Universität Gießen, Gießen, Germany; AG Integrative Sensory Physiology, Institute for Animal Physiology, Justus-Liebig-Universität Gießen, Gießen, Germany

**Keywords:** Insect, Neuroanatomy, Neuronal innervation, Defence glands, Stick insect, Neuronal tracing, Motoneuron, Defence behaviour

## Abstract

**Background:**

Stick insects (Phasmatodea) use repellent chemical substances (allomones) for defence which are released from so-called defence glands in the prothorax. These glands differ in size between species, and are under neuronal control from the CNS. The detailed neural innervation and possible differences between species are not studied so far. Using axonal tracing, the neuronal innervation is investigated comparing four species. The aim is to document the complexity of defence gland innervation in peripheral nerves and central motoneurons in stick insects.

**Results:**

In the species studied here, the defence gland is innervated by the intersegmental nerve complex (ISN) which is formed by three nerves from the prothoracic (T1) and suboesophageal ganglion (SOG), as well as a distinct suboesophageal nerve (*Nervus anterior* of the suboesophageal ganglion). In *Carausius morosus* and *Sipyloidea sipylus*, axonal tracing confirmed an innervation of the defence glands by this *N. anterior* SOG as well as *N. anterior* T1 and *N. posterior* SOG from the intersegmental nerve complex. In *Peruphasma schultei*, which has rather large defence glands, only the innervation by the *N. anterior* SOG was documented by axonal tracing. In the central nervous system of all species, 3-4 neuron types are identified by axonal tracing which send axons in the *N. anterior* SOG likely innervating the defence gland as well as adjacent muscles. These neurons are mainly suboesophageal neurons with one intersegmental neuron located in the prothoracic ganglion. The neuron types are conserved in the species studied, but the combination of neuron types is not identical. In addition, the central nervous system in *S. sipylus* contains one suboesophageal and one prothoracic neuron type with axons in the intersegmental nerve complex contacting the defence gland.

**Conclusions:**

Axonal tracing shows a very complex innervation pattern of the defence glands of Phasmatodea which contains different neurons in different nerves from two adjacent body segments. The gland size correlates to the size of a neuron soma in the suboesophageal ganglion, which likely controls gland contraction. In *P. schultei*, the innervation pattern appears simplified to the anterior suboesophageal nerve. Hence, some evolutionary changes are notable in a conserved neuronal network.

**Electronic supplementary material:**

The online version of this article (doi:10.1186/s12983-015-0122-0) contains supplementary material, which is available to authorized users.

## Introduction

Adaptations for predator avoidance, escape, and active defence have evolved in abundance amongst animals. They increase the potential for survival and thus contribute to the individual fitness of an organism. Two modes of defence are commonly distinguished: primary defence mechanisms (avoiding detection or contact, e. g. by crypsis) and secondary defence mechanisms (defence elicited only after detection or contact) [1, 2]. Secondary defence may include visual displays, secretion of chemicals, or spontaneous production of acoustic signals [2].

Insects use a great variety of primary and secondary defence mechanisms [2–8]. Stick insects (Phasmatodea) are named for their mimetic body shapes, resembling plant elements to avoid detection in the first place. But they are also known for different means of secondary defence, including defence by secretion of chemicals (allomones) [9–12]. The defence secretions are produced by exocrine glands which are located in the prothoracic segment [9, 11]. These glands are an autapomorphy of the taxon Phasmatodea [13, 14]. Secretion of chemicals is an effective defence in stick insects against predators like birds or mammals [9, 10, 15–17] and already nymphs can discharge their glands (*P. schultei* [18]; *S. sipylus* [16]; *O. peruana* [19]). The defence secretion from several species of stick insects has been analysed in detail for components and chemical substances [17, 20–27]. In *P. schultei*, the secretion contains glucose and peruphasmal, a unique dolichodial stereoisomere and the corresponding diol [22].

So far, the defence glands of stick insects have been studied in few species for their functional morphology. The glands produce, store and spray chemical deterrents, and consist of large compressor muscles lined with secretory epithelium [15, 19, 21, 28–32]. They release secretions from the gland through a smaller ejaculatory duct with an opening in the anterior prothorax just behind the head [15, 19, 21, 29, 32, 33]. In *O. peruana*, the large glands extend internally into the mesothoracic segment [19]. The studies on defence gland morphology were carried out on disparate species of stick insects, but variation in sizes and the structure of glands between species are apparent [21, 32].

Amongst stick insects, the only species so far investigated for nerves supplying the defence glands is *Carausius morosus* (Sinety 1901): several innervating nerves have been mentioned including the transverse nerve (*Nervus transversus*) by Marquardt [34]. This study also suggests that the defence glands are innervated by the intersegmental nerve complex including the *Nervus posterior* of the suboesophageal ganglion, *Nervus transversus* and *Nervus anterior* of the prothoracic ganglion [34]. The innervation of the defence gland has not been analysed further, nor has it been compared between different species of stick insects. Since defence glands in stick insects vary in their sizes and their role for secretion in chemical defence (e. g. [11, 21]), this raises the question how similar and conserved the innervation pattern is among stick insects, or if possibly differences occur related to gland sizes. We have studied four species of stick insects from different groups, which also have defence glands of different sizes [31, 32, 34] (Lonchodinae: *C. morosus*; Pseudophasmatinae: *P. schultei*; Necrosciinae: *S. sipylus*; Stephanacridini: *E. tiaratum*). We employed axonal tracing to study the nerves and also motor neurons in the central nervous system which innervate the defence glands.

## Results

### Innervation of defence glands

**Fig. 1:**
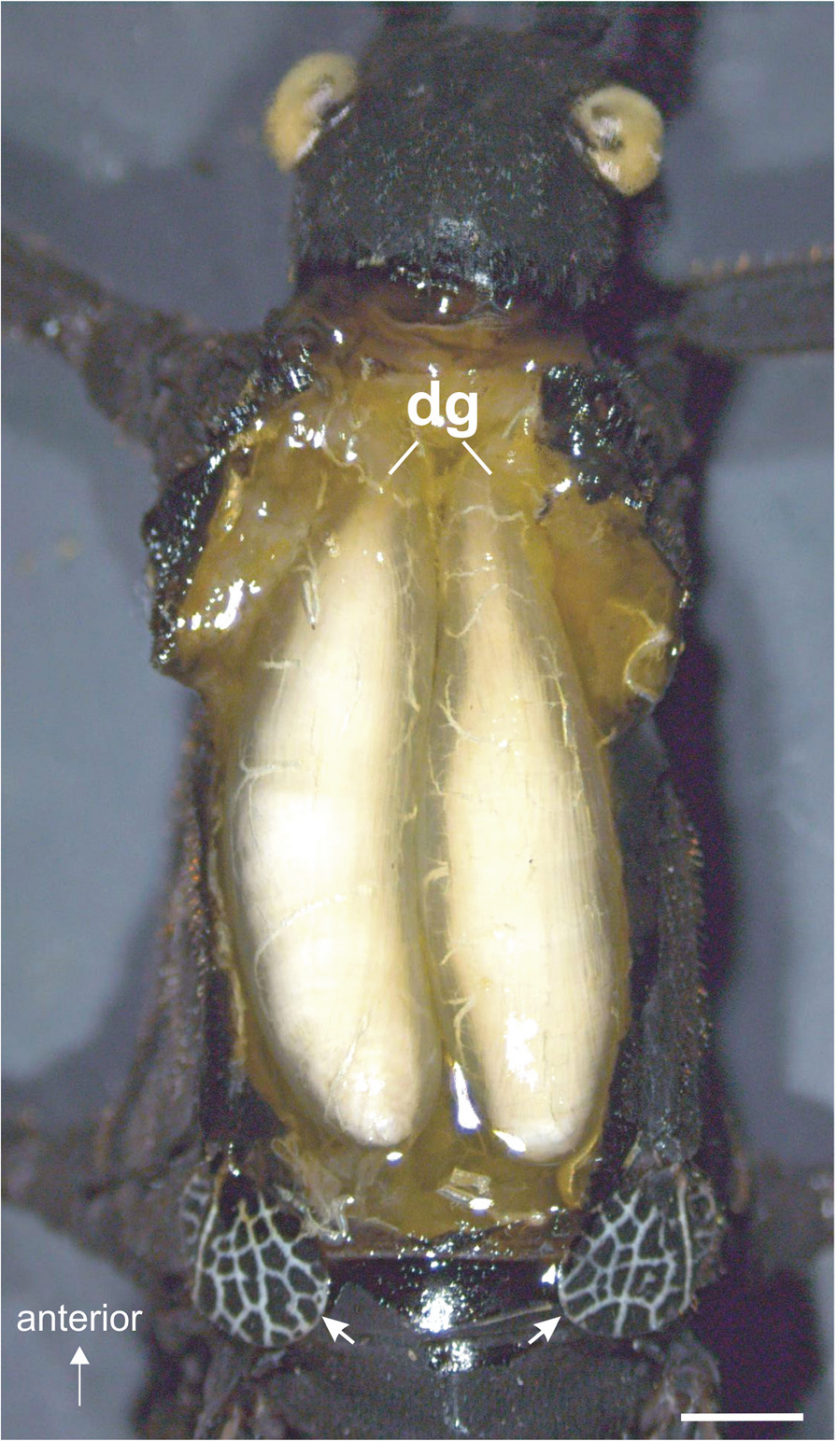
Defence glands in *Peruphasma schultei*. Paired defence glands (dg) in the dorsal prothorax extend into the mesothorax, indicated by the forewing pads (*white arrow heads*). Glands are exposed in an adult male individuum prior to tracing experiments. Scale bar: 1 mm

This study investigates the innervation of defence glands in stick insects in a comparative approach. The defence glands in stick insects vary in size between species. In *P. schultei*, the prothoracic defence glands are similar to other stick insects but are of rather large size and extend into the mesothorax (Fig. 1).

**Fig. 2:**
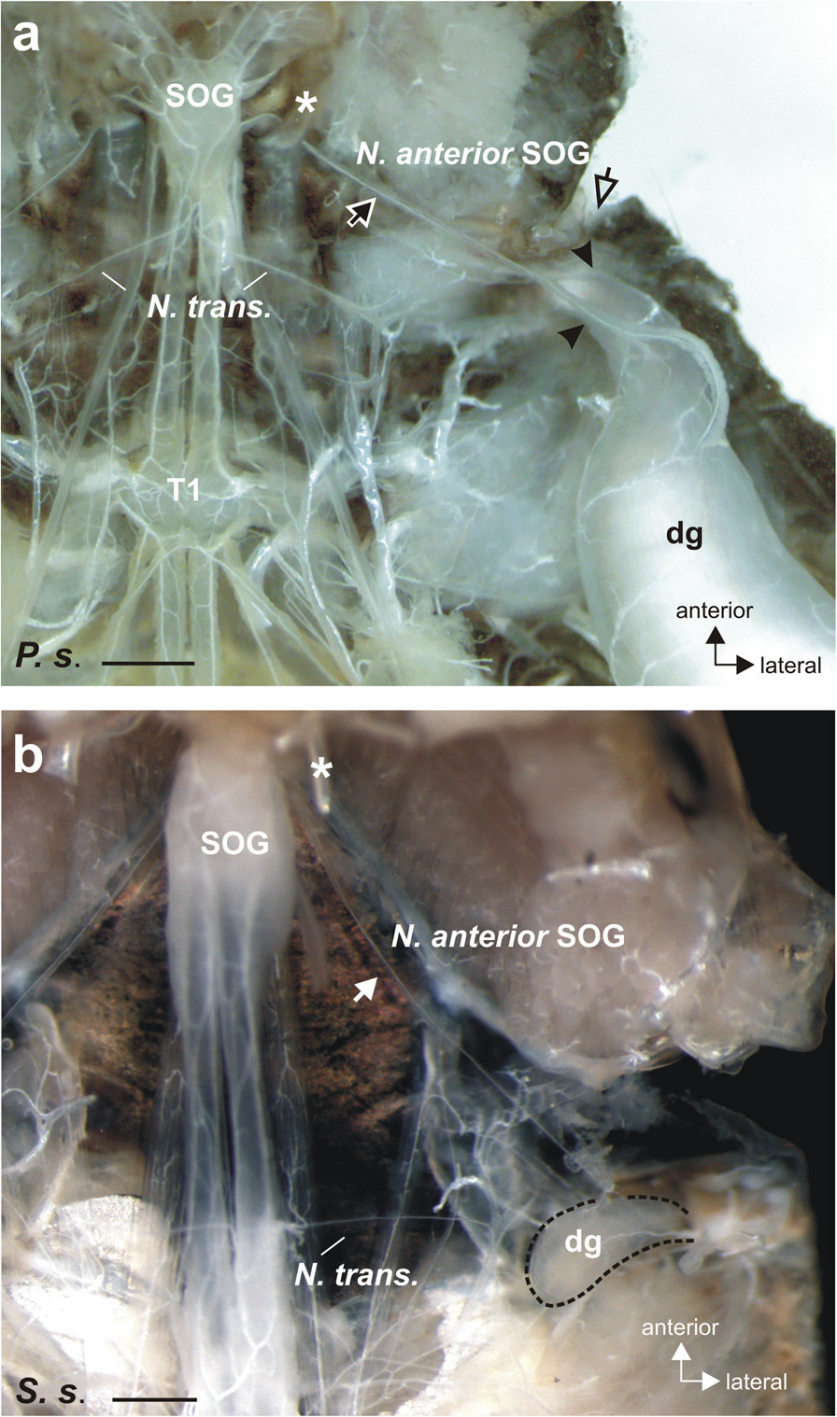
**a** The defence gland in *P. schultei* is innervated by the *Nervus anterior* from the suboesophageal ganglion (SOG) (*filled arrow*) which runs under a cuticular apodeme (*asterisk*) to the ipsilateral gland. The nerve splits into two branches (indicated by two *arrowheads*) on the lateral and dorsal side of the gland. The *empty arrow* indicates the location of the defence gland opening in the thorax. Preparation of an adult female individual. **b** In *S. sipylus*, the defence gland (indicated by *dotted line*) is notably smaller than in *P. schultei*. Innervation also occurs by the *N. anterior* SOG (white arrow). Preparation of an adult female. Abbreviations: dg, defence gland; N. trans., *Nervus transversus*; SOG, suboesophageal ganglion; T1, prothoracic ganglion. Scale bars: (**a**) 1 mm, (**b**) 500 μm

We first studied the nerves supplying the defence glands neuroanatomically in dissected specimen. The most prominent innervation identified is a nerve from the suboesophageal ganglion to contact the ipsilateral gland (Fig. 2a). According to the terminology of nerves by Marquardt [34], this nerve is referred to as the *Nervus anterior* of the suboesophageal ganglion (*Nervus anteror* SOG). This nerve runs ventrally on either side of the ganglion between a cuticular apodeme and the head capsule before entering the prothoracic segment and contacting the defence gland (Fig. 2a, b).

**Fig. 3:**
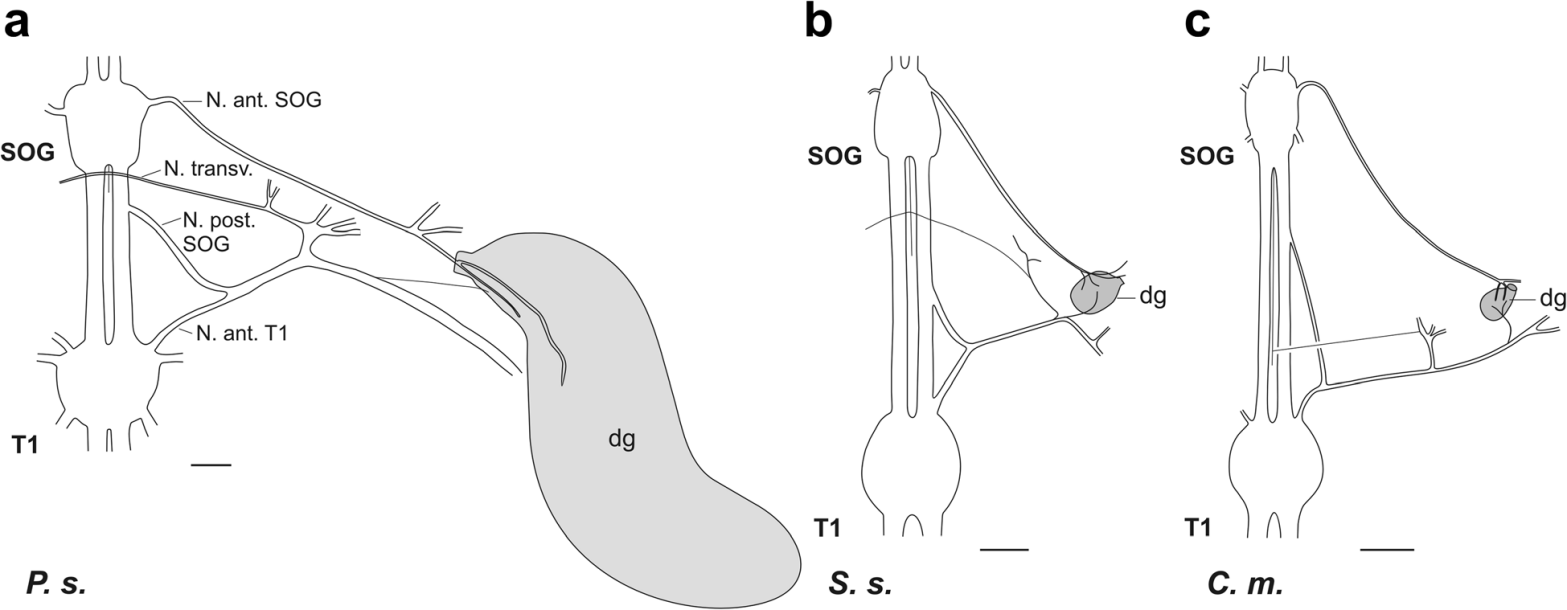
Schematic drawings of the nerves contacting the prothoracic defence gland from *in* *situ*-preparations in **a** *P. schultei*, **b** *S. sipylus*, and **c** *C. morosus*. The nerves have several additional side branches, shown as open endings, for which the innervation targets are not depicted. In *S. sipylus*, two nerve branches from the intersegmental nerve complex of *N. posterior* SOG and *N. anterior* T1 may contact the defence gland. The *N. anterior* SOG and the intersegmental nerve complex also innervate ventral longitudinal muscles by short branches omitted here, before they merge laterally of the defence gland. Nerves are shown for the right side only. Abbreviations: dg, defence gland; N. ant. SOG, *Nervus anterior* SOG; N. ant. T1, *Nervus anterior* T1; N. posterior SOG, *Nervus posterior* SOG; N. trans., *Nervus transversus*; SOG, suboesophageal ganglion; T1, prothoracic ganglion; All scale bars: 100 μm

Additional nerves were noted to run closely to the gland in the prothorax by neuroanatomical investigation in situ (Fig. 3): the *Nervus posterior* of the suboesophageal ganglion and the *Nervus anterior* of the prothoracic ganglion form a joined nerve linked to the glands. This nerve is further laterally also joined by the *Nervus transversus* from the suboesophageal ganglion (together forming the intersegmental nerve complex of Marquardt [34]). The *Nervus anterior* T1 also innervates ventral longitudinal muscles in the prothorax by very short extensions [34]. The *N. anterior* T1 directly merges with the *N. posterior* SOG, without splitting into two separate branches after leaving the ganglion. This simpler organisation differs from the neuroanatomy in the mesothorax, where the *Nervus anterior* T2 splits into two longer branches of which one (na_1_) contacts the *N. posterior* of the prothoracic ganglion, while na_2_ innervates dorsal longitudinal and heart muscles [34–36]. In the prothorax, the three nerves in the intersegmental nerve complex of the prothorax make no individual contacts to the gland (Fig. 3). The connection of nerves in the prothoracic segment is similar between *P. schultei*, *S. sipylus*, and *C. morosus* (Fig. 3a–c) and *E. tiaratum* (not shown). In *P. schultei* and *E. tiaratum*, we did not find differences between the sexes in nerve innervation pattern or the types of innervating neurons in the CNS (see below). The length of individual nerves and the location of their contacts showed some variability between individuals even from the same species. The contact of the intersegmental nerve complex (*N. anterior* T1/ *N. posterior* SOG/ *N. transversus*) to the defence gland is via one short nerve branch or two short branches (Fig. 3; see the two branches in Fig. 6f for *S. sipylus*). Additional branches leave this joined nerve at different locations which innervate thoracic muscles. The defence gland is contacted by a side branch or branches of the joint nerve, while the nerve extends further laterally (Fig. 3). Lateral to the defence gland, the nerve from the intersegmental nerve complex merges with the extension of the *N. anterior* SOG [34].

**Fig. 4:**
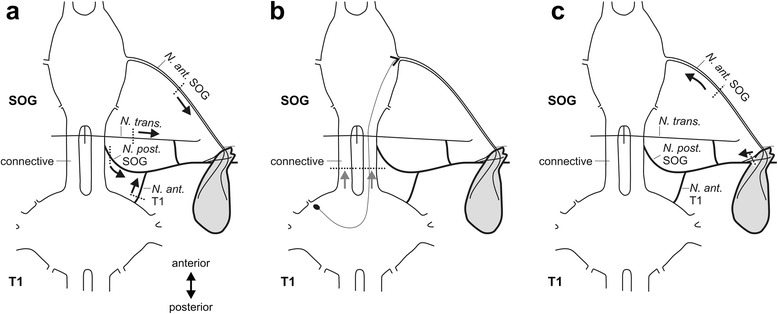
Schematics showing the different nerves studied in axonal tracing experiments. *Dotted lines* indicate the positions where nerves were cut for tracing experiments. *Black arrows* indicate the direction in which axons were filled. **a** Anterograde tracing of nerves to investigate the innervation of the defence gland by nerves from the suboesophageal ganglion (SOG) and prothoracic ganglion (T1). Only one nerve at a time was cut and filled in individual preparations. **b** Anterograde tracing of whole connectives between suboesophageal ganglion (SOG) and prothoracic ganglion (T1) to test for the innervation of defence glands by prothoracic neurons through the *N. anterior* SOG. Soma position and axon of a prothoracic neuron is indicated for one neuron (occurring bilaterally). The connectives were cut posteriorly to the suboesophageal ganglion and then filled simultaneously in anterograde direction, indicated by grey arrows. **c** Retrograde tracing to reveal the neurons innervating the defence gland through the intersegmental nerve complex. The thin nerve branches of the joint *N. posterior* SOG, *N. anterior* T1 and *N. transversus* were cut close to the defence gland. N. anterior SOG was cut midway between ganglia and the glands

The innervation of the glands from the intersegmental nerve complex can only be resolved by neuronal tracing of individual nerves. Besides the *N. anterior* SOG, we therefore tested the *N. posterior* SOG, *N. transversalis*, and *N. anterior* T1 in separate tracing experiments in anterograde direction (for details on the preparations for tracing, see Fig. 4a).

**Fig. 5:**
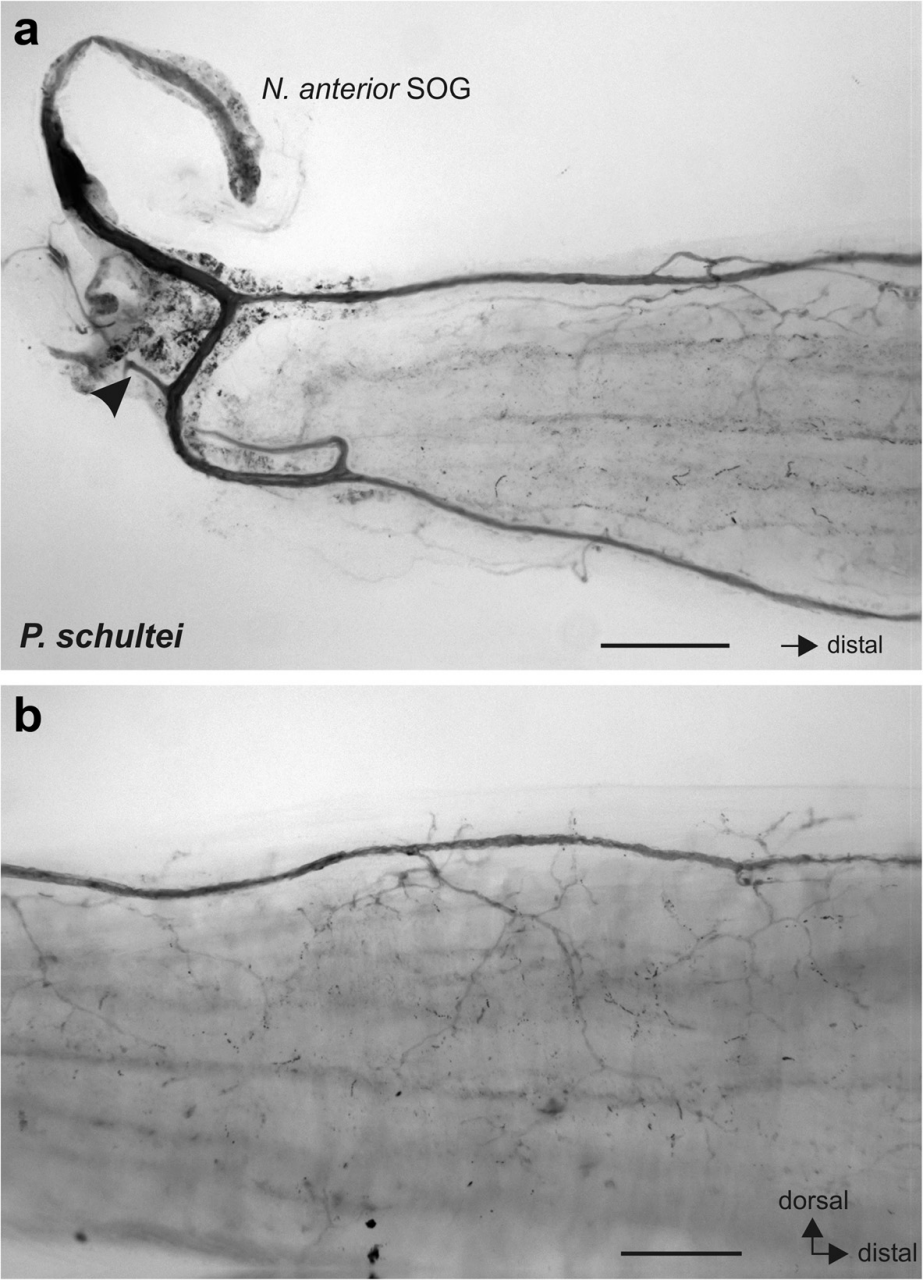
Innervation of the defence gland in *P. schultei* by *N. anterior* SOG as revealed by anterograde tracing with cobalt solution. **a** Anterior part of the defence gland contacted by two nerve branches of the filled *N. anterior* SOG. *Arrowhead* indicates an additional nerve branch innervating adjacent muscles but not the gland. **b** Section from the middle part of the defence gland. Scale bars: (**a**) 250 mm; (**b**) 100 μm

**Fig. 6:**
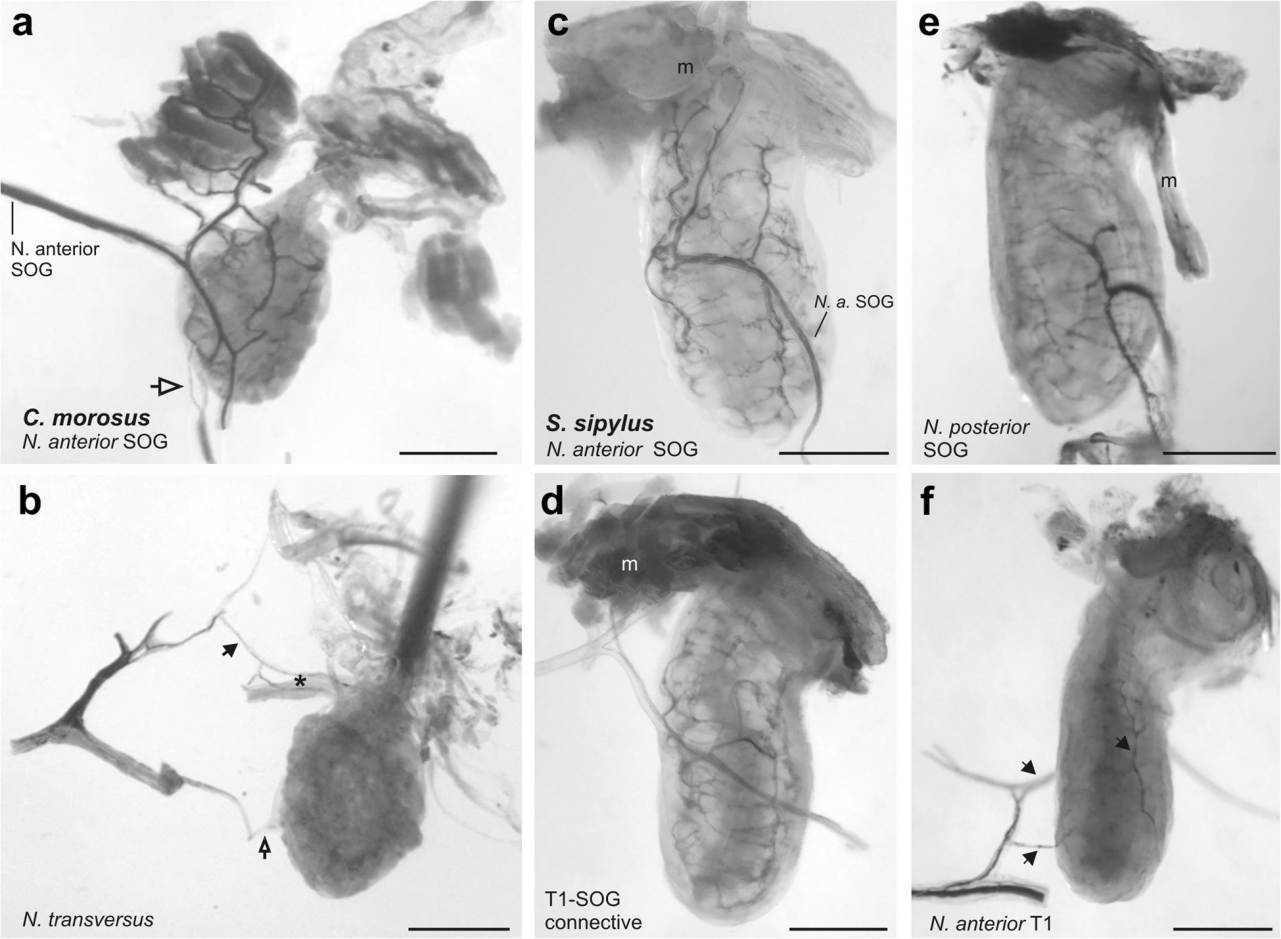
Innervation of the defence gland by different nerves in *C. morosus* (**a**, **b**) and *S. sipylus* (**c**–**f**). In *C. morosus*, tracing of *N. anterior* SOG reveals the innervation of the defence gland and also adjacent muscles. Also visible is the nerve branch from the *N. posterior* SOG/ *N. anterior* T1 which is not stained (*empty arrow*). **b** Tracing of *N. transversus* does not result in staining of nerve fibres on the gland but of a small muscle (*asterisk*) next to the gland by a thin nerve branch (*black arrow*). The nerve branch from *N. transversus* to the gland is not stained (*empty arrow*). Scale bars: 500 μm. In *S. sipylus*, the gland innervation shown by neuronal branches on the gland surface from several nerves: **c** the *N. anterior* SOG, **d** the *N. anterior* SOG via the prothoracic-suboesophageal connective, **e** the *N. posterior* SOG, and **d** the *N. anterior* T1. In all preparations neurobiotin solution was used as tracer. Scale bars: 500 μm

Anterograde neuronal tracing allows to reveal the innervation of the defence glands by the *N. anterior* SOG (Figs. 5, 6a, c). In *P. schultei*, *N. anterior* SOG innervates the gland after splitting into two branches by both of these branches (Figs. 2a, 5). Multiple fine neurites split off the two branches to contact the surface of the gland (Fig. 5b). Similar results were obtained by tracing the *N. anterior* SOG in *C. morosus* (Fig. 6a) and *S. sipylus* (Fig. 6c). These findings confirm that the suboesophageal ganglion directly innervates the defence glands via the *N. anterior* SOG. Tracing the connectives between prothoracic and suboesophageal ganglion (Fig. 4b) showed a very similar innervation pattern on the defence gland, indicating an intersegmental innervation through the *N. anterior* SOG from the prothoracic ganglion (Fig. 6d; see below on the source of this innervation).

In the intersegmental nerve complex, the *N. posterior* SOG also innervates the defence gland in *S. sipylus* (Fig. 6e) and *C. morosus* (not shown). From the prothoracic ganglion, *N. anterior* T1 innervates the glands in *S. sipylus* (Fig. 6f) and in *C. morosus* (not shown). *N. anterior* T1 innervates the defence gland by short nerve branches shared with the *N. posterior* SOG (Fig. 3a–c), and these also innervate other muscles in the prothorax (Fig. 6e, f). This innervation of the defence gland by *N. anterior* T1 was also found in *C. morosus* (not shown). However, the tracing of *N. posterior SOG* or *N. anterior* T1 did not reveal an innervation on the defence gland in *P. schultei* (not shown). A direct contact of *N. transversus* to the glands could not be detected by dissection in any species studied (for overview, see Fig. 3a–c). Neuronal tracing of the *N. transversus* in *P. schultei*, *C. morosus* and *S. sipylus* revealed innervation of a small muscle in the prothorax next to the defence gland, but not of the defence gland itself (Fig. 6b for *C. morosus*). The different nerves innervating the defence glands are summarised in Table 1.

**Table 1:** Nerves innervating the defence glands in four stick insect species

	*Peruphasma schultei*	*Sipyloidea sipylus*	*Carausius morosus*	*Extatosoma tiaratum*
*N. anterior* SOG	+	+	+	+
*N. posterior* SOG	-	+	+	n. d.
*N. transversus*	-	-	-	n. d.
*N. anterior* T1	-	+	+	n. d.

*N. posterior* SOG, *N. transversus* and *N. anterior* T1 form the intersegmental nerve complex

*n. d* not determined

An activation of the gland by different nerves was tested by applying electrical stimuli to the nerve in *P. schultei*. Muscle contractions were elicited by stimulation of the *N. anterior* SOG with stimuli starting from 0.3 V amplitude and 5 ms duration (*n* = 5), thus conforming the innervation and neuronal control of the gland by this nerve. Stimulation of the connective (to activate the PIN neuron axon) did also lead to a gland contraction (*n* = 3). Stimulation of the *N. transversus* did not result in contraction of the gland but of other lateral muscles (*n* = 5). The stimulation of nerves in the intersegmental nerve complex did not contract the defence gland (*n* = 8), which is consistent with the lacking innervation found in tracing experiments.

### Central neurons with axons in gland innervating nerves

**Fig. 7:**
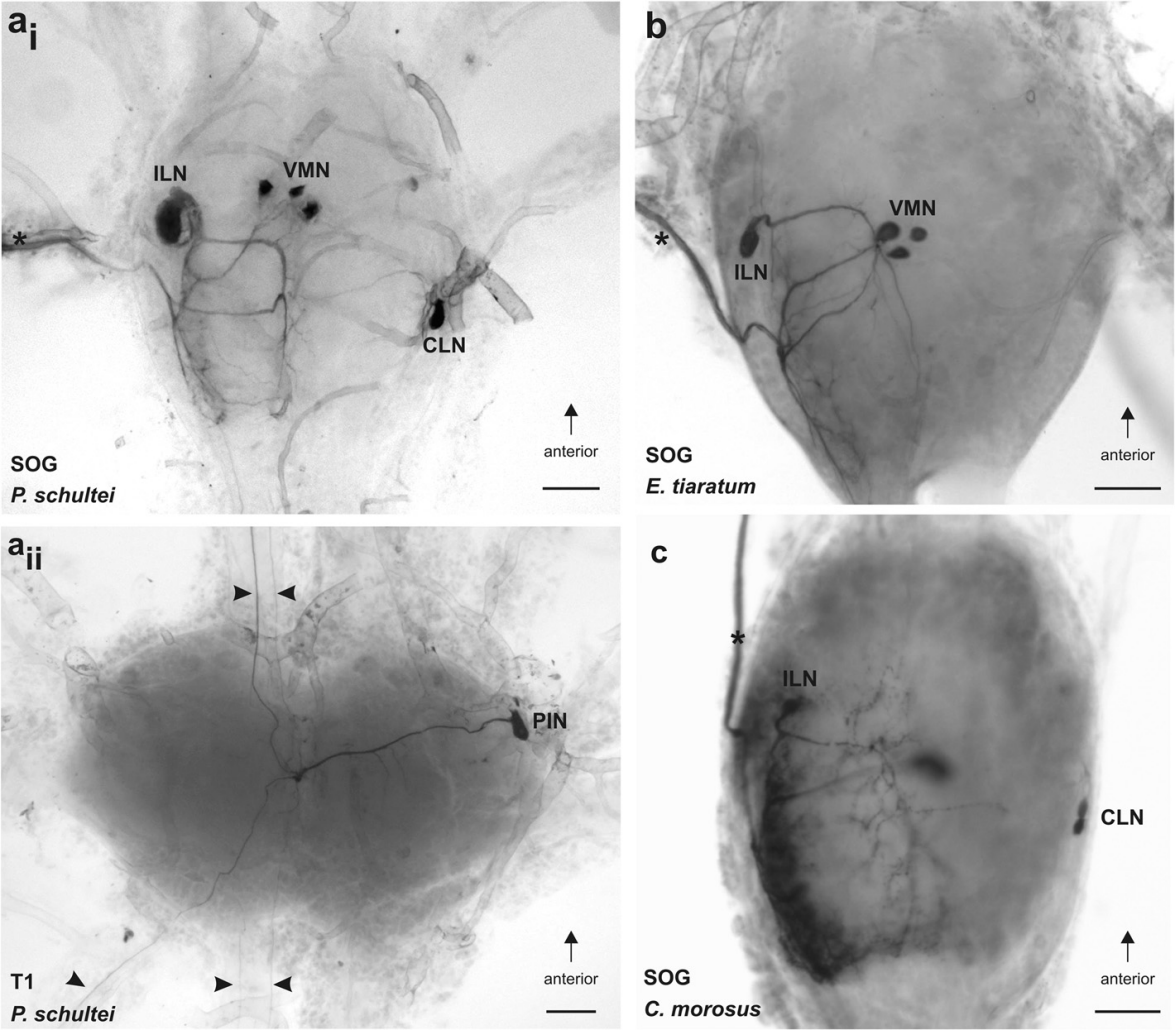
Backfill preparations with neurons supplying the *N. anterior* SOG in the suboesophageal and prothoracic ganglia. The filled nerve *N. anterior* SOG is indicated by asterisks. Stained fibres in the connectives and peripheral nerves of *P. schultei* are indicated by arrowheads. Preparations of **a**_i-ii_ *P. schultei* are from a male individual, and of **b** *E. tiaratum* from a female, and **c** *C. morosus* from a female. In all preparations neurobiotin solution was used as tracer. Abbreviations: CLN, contralateral neurons; ILN, ipsilateral neuron; PIN, prothoracic intersegmental neuron; SOG, suboesophageal ganglion; T1, prothoracic ganglion; VMN, ventral medial neurons. All scale bars: 100 μm

**Fig. 8:**
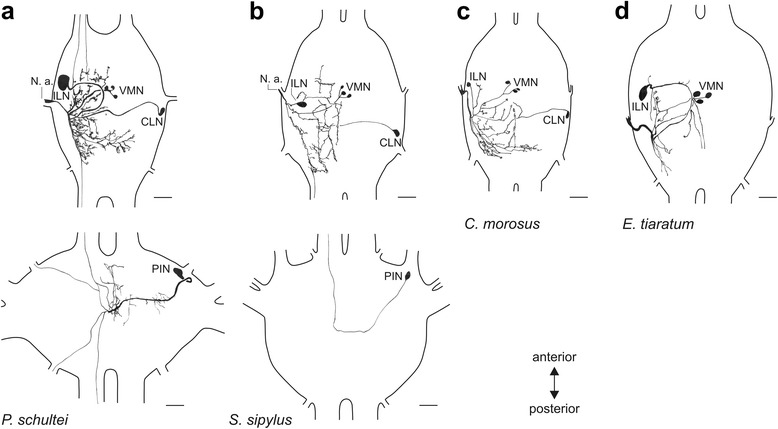
Distribution of neurons in the suboesophageal (*top row*) and prothoracic ganglion (*bottom row*) revealed by retrograde tracing of the left *N. anterior* SOG in **a** *P. schultei*, **b** *S. sipylus*, **c** *C. morosus*, and **d** *E. tiaratum*. Ganglia are shown in dorsal (top) view after filling of the left *N. anterior* SOG. The nerves in all original ganglia preparations were traced using neurobiotin solution as tracer. Abbreviations: CLN, contralateral neuron; ILN, ispilateral neuron; N. a., *Nervus anterior* SOG; PIN, prothoracic neuron; VMN, ventral median neurons. All scale bars: 100 μm

**Fig. 9:**
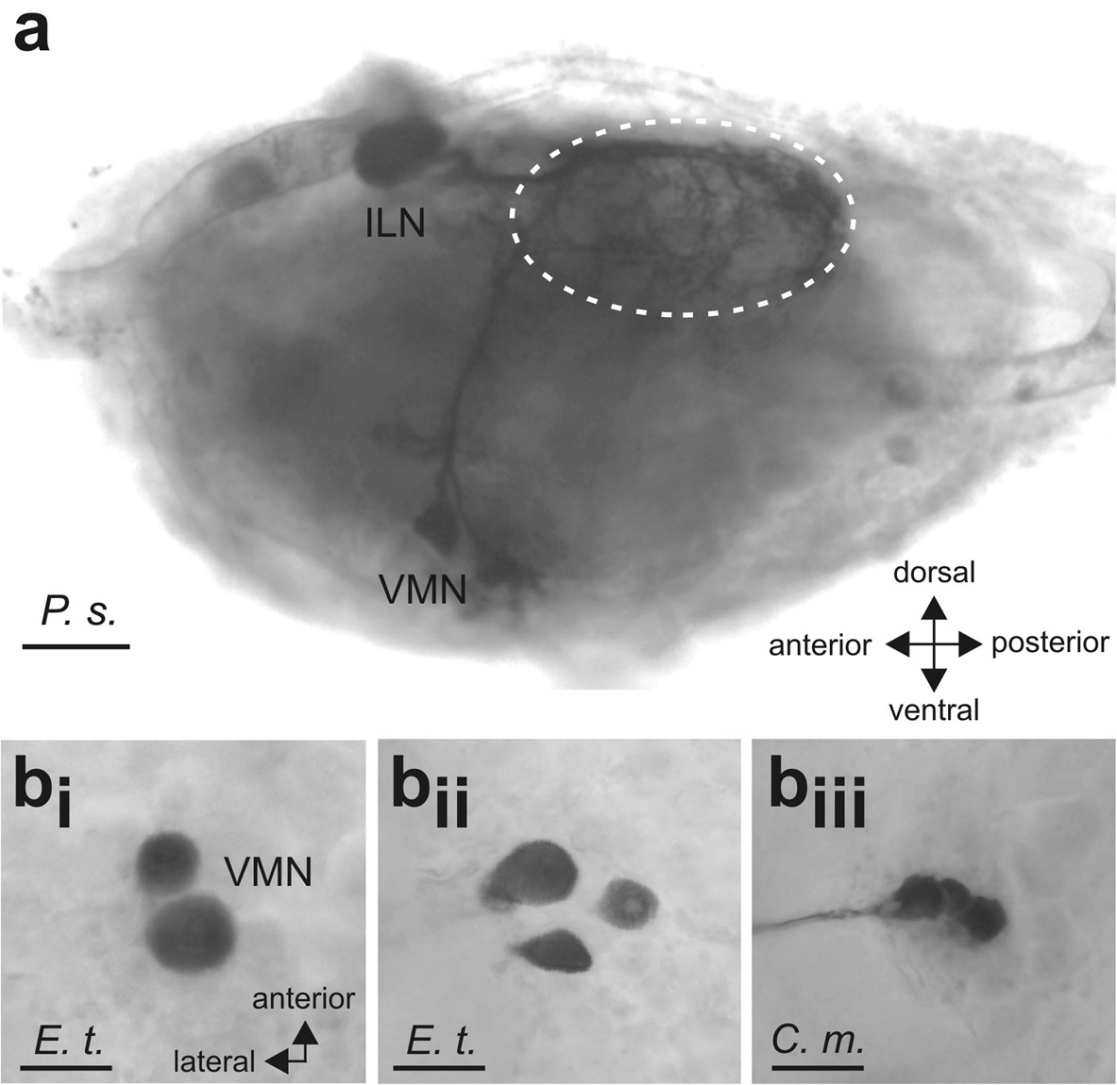
**a** Lateral view on the suboesophageal ganglion of *P. schultei* showing the ILN and VMNs. Dotted circle outlines the dorsal area of neurites. **b**_i-iii_ The number of VMNs is variable, as shown with 2 (*b*_*i*_), 3 (*b*_*ii*_) or 4 (*b*_*iii*_) neurons. Preparations of *E. tiaratum* from females. The nerves in all original ganglia preparations were traced using neurobiotin solution as tracer. Abbreviations: ILN, ipsilateral neuron; VMN, ventral median neurons. Scale bars: (**a**), 100 μm; (*b*_*i*–*iii*_), 50 μm

Retrograde tracing of the *N. anterior* SOG in all four species of stick insects (Fig. 4c) stained neurons in the suboesophageal ganglion (Figs. **7**, 8; Table 2). Consistently, a neuronal cell body in the anterior SOG was stained ipsilateral to the filled nerve, as were 2–5 smaller cell bodies located at the ventral midline in all four species studied (Figs. **7**a_i_, b, c; 8; 9b; Table 2). The small neurons are located at the ventral side of the ganglion, while the ipsilateral neuron is located in a dorsal position (Fig. 9a). These types of neurons were termed according to their position ipsilateral neuron (ILN) and ventral medial neurons (VMN). In *P. schultei*, *C. morosus*, and *S. sipylus*, a single neuron located on the contralateral side is present (Figs. **7**a_i_, c; 8). This neuron is referred to as contralateral neuron (CLN). Its soma is located in a medial position in the ganglion in the ventro-lateral axis. The CLN soma is generally located more posteriorly in the ganglion than the soma of the ILN (Figs. ***7***, 8). In *C. morosus*, in one specimen two CLNs were found (Fig. 7c). This neuron was not found in *E. tiaratum* (Fig. 7, 8c; *n* = 6), and is therefore assumed to be absent in this species.

**Table 2:** Types of neurons, numbers, and their localisation as identified by tracing the nerves innervating the defence glands of stick insects. In all tracing experiments, neurobiotin solution was used unless indicated otherwise

	*Nervus anterior* SOG		*Nervus posterior* SOG/*Nervus anterior* T1 (Intersegmental nerve complex)	
	SOG	T1	SOG	T1
*Carausius morosus*	1 ILN, 1-2 CLN, 3-4 VMNs (*n* = 9)	- (*n* = 24)	n. d.	n. d.
*Sipyloidea sipylus*	1 ILN, 1 CLN, 2-5 VMNS (*n* = 25)	1 PIN (*n* = 25)	1 SMN (*n* = 20)	1-2 DUM, 1 DN (*n* = 20)
*Peruphasma schultei*	1 ILN, 1 CLN, 3 VMNs (*n* = 24: *n* = 6 traced with cobalt solution; *n* = 18 traced with neurobiotin solution)	1 PIN (*n* = 24)	n. d.	n. d.
	Tracing of *N. ant*. SOG to brain: *n* = 8			
	Tracing of *N. ant*. SOG to T2/T3: *n* = 8			
*Extatosoma tiaratum*	1 ILN, 2-3 VMNs (*n* = 6)	- (*n* = 6)	n. d.	n. d.

*ILN* ipsilateral neuron, *CLN* contralateral neuron, *DN* dorsal neuron, *DUM* dorsal unpaired median neurons, *n. d* not determined, *PIN* prothoracic intersegmental neuron, *SMN* suboesophageal midline neuron *SOG*, suboesophageal ganglion, *VMN* ventral median neuron

In addition, retrograde tracing of *N. anterior* SOG revealed a single neuron soma in the prothoracic ganglion in *P. schultei* (Figs. 7a_ii_, 8a) and *S. sipylus* (Fig. 8b). This soma occurs on the contralateral side to the *N. anterior* SOG being filled. One to two ascending fibres in the neck connective indicate processes of this neuron extending into the suboesophageal ganglion (Fig. 8a). This neuron is referred to as prothoracic intersegmental neuron (PIN). We found the PIN in *P. schultei* and *S. sipylus* but never in *C. morosus* or *E. tiaratum* (Table 2). We tested whether the PIN innervates the defence glands by filling the connectives (including the PIN axon running in the connectives; Fig. 4b) between the prothoracic and the suboesophageal ganglion in adult individuals of *P. schultei* and fourth instars of *S. sipylus* (Fig. 4b). These tracing experiments did not reveal any staining of nerves innervating the glands in *P. schultei*, possibly due to the rather long distance between the connectives and the defence glands via *N. anterior* SOG in adult individuals. However, the tracing of connectives in *S. sipylus* instars showed an innervation of the gland similar to the pattern revealed by the tracing of the *N. anterior* SOG (Fig. 6d), confirming that the PIN innervates the defence gland via the *N. anterior* SOG. Supposedly, PIN in *P. schultei* has additional targets, as neurites from the main axon also enter the *Nervus anterior* and *N. posterior* of the prothoracic ganglion (Figs. **7**a_ii_, 8a). Though in *P. schultei* one to two fibres were noted descending further to the mesothoracic ganglion (Figs. **7**a_ii_, 8a), we did never find stained somata in this or the more posterior metathoracic ganglion. Similarly, we noted one to two ascending fibres from the suboesophageal ganglion into the brain in *P. schultei*. We could not detect stained neuron somata in the brain (supraesophageal ganglion), and few fibres with faint ramifications terminate in the ventral deutocerebrum in *P. schultei* (not shown).

The neurites of the suboesophageal neurons innervating the gland via *N. anterior* SOG are concentrated in the ganglion on the ipsilateral side to the filled nerve (Figs. **7**, 8). Here, they locate in the posterior part of the ganglion. Since the neuronal ramifications are rather dense, it was not possible to ascribe them to a particular neuron. They locate mainly to the dorsal part of the ganglion (Fig. 9a).

The number of some neuron types varied between individuals. The ILN was always found as a single neuron, but in *C. morosus*, two closely associated CLNs occurred in a single preparation (Fig. 7c). A higher variability occurred in the VMNs, which occur as two to four neurons (Fig. 9b_i_-b_iii_), and up to five found in *S. sipylus* (see Table 2). In sum, we found neuron types common among different stick insect species, though individual neuron types can be missing in a specific species (PIN, CLN).

**Fig. 10:**
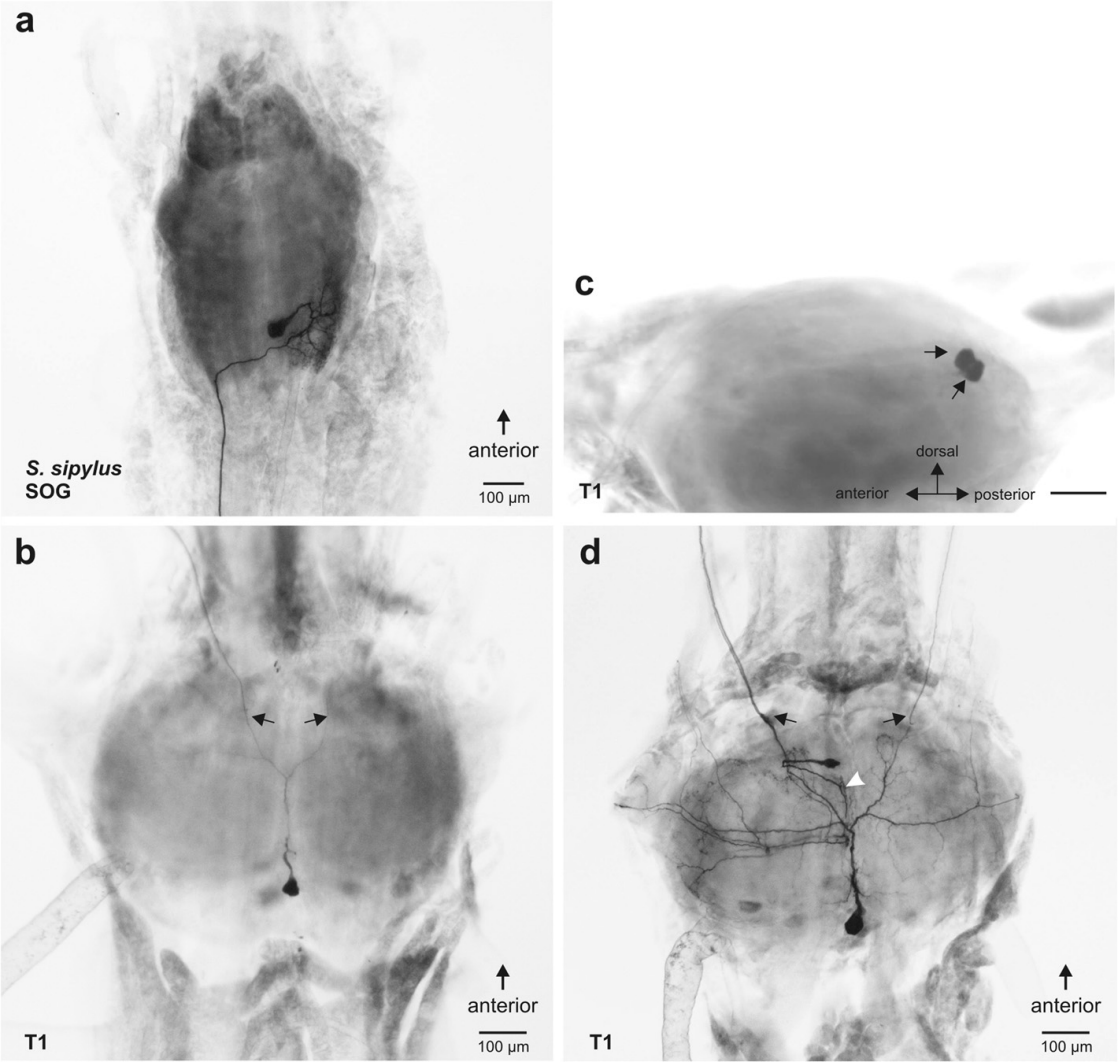
Tracing preparations from *S. sipylus* with neurons innervating the defence gland via the intersegmental nerve complex (*N. posterior* SOG and *N. anterior* T1) in **a** the suboesophageal ganglion and (**b**–**d**) the prothoracic ganglion. In the preparations in dorsal view (**a**–**b**, **d**), the left nerves were filled. Prothoracic dorsal neurons located posterior in the ganglion are DUM neurons with bilaterally symmetrical neurites, see arrows in (**b**, **d**). **c** Lateral view of the prothoracic ganglion reveals two DUM neuron somata in the dorsal ganglion in close contact. A further prothoracic neuron occurs located at the dorsal midline (**d**). The neurite runs medially (*white arrowhead*) in the ganglion. Nerves in the ganglia were traced using neurobiotin solution. Abbreviations: SOG, suboesophageal ganglion; T1, prothoracic ganglion. Scale bars: 100 μm

We also traced the nerve branch or branches from the intersegmental nerve complex (*N. posterior* SOG and *N. anterior* T1) directly contacting the distal gland in *S. sipylus* in retrograde direction (Fig. 4c). These tracings are more difficult to carry out since the branches onto the gland are rather short, while the main nerve also supplies adjacent thorax muscles (Fig. 3). In *S. sipylus*, retrograde tracing stained fibres within both the *N. posterior* SOG and *N. anterior* T1, and revealed a single soma in the suboesophageal ganglion (Fig. 10a) and one to three somata in the prothoracic ganglion on the midline (Fig. 10b). These latter neuron or neurons locate dorsally in the posterior ganglion (Fig. 10c). Somata were generally clearly stained, while the bilateral neurites reveal the form indicative of dorsal unpaired median (DUM) neurons (Fig. 10b, d). Either one (*n* = 11 in 19 preparations) or two neurons (*n* = 8 in 19 ganglia; Fig. 10c) was found. In the prothoracic ganglion, we also stained another neuron with the soma close to the midline (Fig. 10d). This neuron was stained less reliably and was found in 9 of 20 preparations tracing the intersegmental nerve complex (Fig. 10d). In *C. morosus*, the nerve branches to the gland were too small for adequate tracing. In *P. schultei*, the innervation by the intersegmental nerve complex was not tested as the *N. posterior* SOG or *N. anterior* T1 could not be shown to innervate the glands by anterograde tracing (see above, and Table 1).

## Discussion

### Neural innervation of defence glands in stick insects

The most prominent nerve innervating the defence gland in all species studied is the *N. anterior* SOG. In general, the SOG is an important motor centre in insects, in particular for the mouth parts [37–39] but also movement of legs [40, 41], and in grasshoppers it even controls sound production by leg stridulation [42]. The SOG is also involved in the processing of sensory information, including mechanosensory inputs [43–45], and auditory information in locusts [46].

Contrary to the description provided by Marquardt [34], we could not detect a direct innervation of defence glands by the *N. transversus* in *C. morosus* or the other stick insect species where it was studied (*P. schultei*, *S. sipylus*). The intersegmental nerve complex from SOG and T1 innervates the defence glands by the *N. posterior* SOG and the *N. anterior* T1 in *C. morosus* and *S. sipylus* (Table 1; Fig. 6). Distinct neurons running from the respective ganglia project through the joint nerve branch to the defence gland (Fig. 10). Overall, we provide evidence that three nerves innervate the defence gland in *C. morosus* and *S. sipylus*: *N. anterior* SOG, *N. posterior* SOG, and *N. anterior* T1 (summarised in Table 1). The elements of the intersegmental nerve complex in *P. schultei* could not be shown to innervate the gland.

### Identified neurons and their possible innervation of the defence gland

Insect motor neurons can readily be compared between species, as motor innervation patterns and neuron soma position are commonly rather conserved [47–51]. We have identified four neuron types potentially innervating the stick insect defence gland via the *N. anterior* SOG, and one neuron type via *N. posterior* SOG and two types via *N. anterior* T1 potentially innervating the defence gland (Table 2). Across the four species studied, neuron numbers vary between individuals mainly for the VMNs but also an additional CLN in *C. morosus* occured. The CLN was not found in *E. tiaratum*. The prothoracic PIN was found only in *P. schultei* and *S. sipylus*, but never in *C. morosus* and *E. tiaratum*. Hence, the neuron types are conserved in the stick insect species studied here, but their combination may differ somewhat between species.

For nerves innervating the defence glands, the additional terminations on adjacent muscles make it difficult to definitely identify the neurons which actually innervate the defence gland. The ILN is the most prominent SOG neuron projecting into the *N. anterior* SOG in *P. schultei*, *S. sipylus*, and *E. tiaratum* (Figs. **7**, 8). Its soma size appears to correlate to the size of the defence glands (see below), and it likely innervates the gland muscles. The VMNs resemble motoneurons of neck muscles described in locusts and crickets [37, 52], and are thus good candidates for a pre-existing nerve-muscle system that was transformed into a exocrine gland surrounded by compressor muscles, while maintaining the motoneurons and adding further neurons (ILN). The stimulation of *N. anterior* SOG supports the activation of the gland by this nerve. A possible source of falsely stained neuron somata that do not actually innervate the gland would be neurosecretory or peptidergic cells with release sites in the periphery [53, 54] and with extensions possibly located on the nerves traced, including the DUM neurons. This false staining may potentially occur for the PIN and CLN as well as the DUM, especially since the PIN resembles peptidergic CCAP-immunoreactive neurons from locusts [54] and the stick insect *Baculum extradentatum* [55]. However, the gland innervation by the prothoracic PIN neuron in *S. sipylus* is likely since filling of the connectives shows innervation of the gland (Fig. 6d). One functional aspect of parallel innervation by different neurons may be a modulatory function of subsets of neurons. The PIN resembles neurons expressing crustacean cardioactive peptide (CCAP) in locusts [54] in soma position and also neurite morphology. CCAP is present in phasmid species [55, 56], and may modulate gland function. As an alternative, the intersegmental connectivity may allow an activation upon mechanosensory input from the (pro-) thorax, e. g. upon contact like touch or grasping. Apparently, the PIN occurs in the species with relatively strong chemical defence (*P. schultei*, *S. siyplus*), and seems to support gland usage.

At least two neuron types (ILN, PIN) may thus innervate the defence glands via *N. anterior* SOG. An innervation by the VMNs and the CLN is also possible, but not directly shown by tracing experiments or morphological features of neurons. The short nerve branches from *N. posterior* SOG and *N. anterior* T1 were filled very closely to the gland, and revealed a suboesophageal neuron and a prothoracic DUM neuron. DUM neurons are usually modulatory neurons [57]. The broad projections seen in thoroughly stained cells suggest they have several further contact sites. More general, the neurons described here for the intersegmental nerve complex which likely innervate the glands (Fig. 10) may also project to additional muscles, probably controlling the prothoracic opening or the ejaculatory duct.

Some comparison may be drawn to the innervation of the salivary glands in stick insects and locusts, also located in the thorax and innervated by the salivary nerve from the SOG [37, 58, 59]. The salivary nerve innervates the salivary glands through axons of two different neurons: the SN1, located anteriorly on the contralateral side in dorsal position, and the SN2, located more posteriorly on the ipsilateral side in ventral position. These two neurons use different transmitters, dopamine (SN1) or serotonin (SN2), suggesting also different functions in gland control for the neurons. In other insects like locusts, the innervation can be more complex involving unpaired median neurons and additional nerves [57, 58, 60, 61].

### Different control strategies for chemical defence in stick insects

Stick insects use defence secretions generally in two possible ways against predators: the secretions cover their own body surface, or they can spray into the direction of the attacker [9, 11]. For *O. peruana*, both strategies are described [21]. The most prominent neuron type in the suboesophageal ganglion is the single ILN in *P. schultei* and *E. tiaratum*, while this neuron is less prominent in *S. sipylus* and *C. morosus*. These differences may correlate to differences in the use of chemical defence between species: *P. schultei* sprays the secretions [18] and *E. tiaratum* females spray both the attacker as well as themselves [32]. In *S. sipylus*, the defence secretions are used to cover the body [10]. *C. morosus* has relatively small defence glands which are not used for chemical defence [40, 62, 63]. Hence, the conserved set for motoneurons subserves different strategies of how chemical secretion is used for defence.

Different sensory inputs and pathways activate the secretion from defence glands, especially upon direct contact via mechanosensory stimuli as well as visual stimuli ([15, 19, 21, 28–32] for *Anisomorpha buprestoides*). Other modalities such as acoustic sensitivity (as tympanal organs are lacking) or cercal stimuli have not been reported, while the role of antennae or ocelli has not been sufficiently studied. The connectivity could be anatomically correlated to the different neuropiles in the ganglia [64–66]. Further analysis of the neurites localisation, e. g. in neuropiles of the ganglia with mechanosensory inputs [67] might hint at the dominant stimuli inputs to the ILN, VMNs, or the single SOG neuron projecting through the *N. posterior* SOG. However, the densely packed neurites, especially in the suboesophageal ganglion, did not allow to differentiate the fibres from individual neurons.

The strategies to avoid predator attacks vary between the phasmid species studied in primary and secondary defence mechanisms. While *C. morosus* relies mainly on mimicry and thanatosis [40, 62, 68], *E. tiaratum* is not only camouflaged but can also offend potential enemies with spines, occasionally display legs [10, 69], and spray towards their attacker and impregnate themselves with defensive fluid from their defence glands [70]. *S. sipylus* has different strategies for defence: they release their defensive fluid by covering their body, but also have a tendency for thanatosis (e.g. mimicking dry grass, falling onto the ground) [10]. If a leg is pinched, autotomy is induced, and if disturbed further, flight is used over short distances for escape [71]. *P. schultei* behaves in a different manner: disturbed individuals react by curling of the abdomen (probably increasing pressure within the body cavity). However, the role of abdominal curling induced by predators is not clear [10]. The adults also display the bright red alae [18]. Other defence behaviour like mimicry or autotomy is not used. If disturbed persistently, *P. schultei* release defensive secretions from their defence glands, and become more motile. Evasion by dropping to the ground is observed mainly in larval instars. Thus, the chemical defence is the only highly important line of defence in *P. schultei*. In sum, this suggests that the ILN size may correlate to the release of defensive spray. It is not immediately evident why the functional specialisations of the neurons should show a correlation in cell body sizes. However, in grasshoppers (*Barytettix psolus*; Acrididae: Melanoplinae) which lost wings and flight during evolution, the flight motoneurons persisted but show a reduction in soma size compared to flying locusts [48]. It would hence be interesting to extend the neuroanatomical study of the ILN between further stick insect species with different modes of defensive secretion. In addition, the neurons innervating the defence glands in *Timema* species would be of interest, as Timematodea are the basal lineage in Phasmatodea and the sister group to Euphasmatodea [13], and a defensive repellent function of gland secretions is not established so far [72].

### Homology of gland innervation elements in stick insect to neuronal elements in Orthoptera and Blattodea

Neuronal systems similar to the defence gland innervation in stick insects have been described for the neck muscle system of Orthoptera [37, 52] and cockroaches [73]. The *N. anterior* SOG is presumably homologous to the suboesophageal nerve 6 of locusts and crickets [37, 52] and the suboesophageal tergal nerve of cockroaches [73].

In particular for these suboesophageal nerves, neurons homologous to some types of stick insect neurons have been identified (references above, and Additional file 1: Table S1). The VMNs of stick insects are a ventral group of 2–4 neurons which is similarly positioned in the other insect orders mentioned above (Additional file 1: Table S1), the DL1 neurons of cockroaches [73] and the neurons innervating the dorsal longitudinal muscles 50/ 51 via nerve 6 in locust and crickets [37, 52]. In these insects, the respective neurons innervate neck muscles, which suggests that the defence glands most likely derive from prothoracic longitudinal neck muscles 50/ 51 of ancestors of these taxa [37, 52]. This hypothesis could be tested further with respect to the sets of muscles present in these insects. However, the stick insect system obviously has some specialisations in neurons which have no counterparts in Orthoptera, in particular the prominent ILN (Additional file 1: Table S1). The ILN may thus have evolved together with the larger compressor muscle of the defence gland in stick insects, and therefore lacks a homologous neuron in Orthoptera.

For some neurons with axons in the intersegmental nerve complex, homologous neurons are more difficult to identify across orthopteroid insects than those in the *N. anterior* SOG. One reason may be the apparent differences in neurite patterns and exact soma position. For the SOG neuron, possible homologous cells have been described in crickets [52] (a single SOG neuron also innervating neck muscles 50/ 51 via N8) and cockroaches [73] (DL_3_ neurons which also innervates dorsal longitudinal (DL) muscle). The 1-2 prothoracic DUM neurons are identified by the bilaterally symmetric neurites. They are homologous to the DUM neuron in the cockroach [73] and locust [52] also supplying neck muscles.

In summary, for the stick insect VMNs, clearly homologous motoneurons exist in Orthoptera (locusts and crickets) and the cockroach (*Periplaneta*), and likely also for the ISN with the suboesophageal neuron and the prothoracic DUM neuron pair. The prominent ILN appears to be unique to stick insects.

## Conclusion

We report here that the defence glands in three species of stick insects (*C. morosus*, *S. sipylus*, *E. tiaratum*) are innervated in a complex way by three nerves, two of these merge in the intersegmental nerve complex. These nerves originate in the suboesophageal and the prothoracic ganglion. In *P. schultei*, only the *N. anterior* SOG innervates the defence glands. The *N. transversus* however does not innervate the defence glands in the species studied. In the central nervous system, we found motoneurons differing in morphology which may innervate the defence glands. Three of four neuron types locate in the suboesophageal ganglion. Remarkably, the forth type of neuron in *P. schultei* and *S. sipylus* has a cell body located in the prothoracic ganglion and sends fibres into the suboesophageal ganglion and via the *N. anterior* SOG to the glands. We find differences between the species in combinations of neurons and also differences in the morphology of one suboesophageal neuron (ILN). The ILN soma size correlates to the biology of the species regarding their mode of chemical defence. This suggests that the ILN is a motoneuron controlling gland contraction. The VMNs are homologous to motoneurons commonly innervating neck muscles in crickets and grasshoppers, and can be supposed to also innervate the gland in stick insects. The PIN is probably a modulatory neuron contacting the gland, while the role of the CLN is less clear. The neurons found in the intersegmental nerve complex include one consistently stained motoneuron and neuromodulatory DUM neurons in the prothoracic ganglion. It has to be kept in mind that probably some of the neuron types innervate also in addition, or even exclusively, other thoracic muscles than the gland. Accordingly, the most likely candidates for gland innervation are the ILN and VMN neurons.

## Materials and methods

### Stick insect rearing

Four species of stick insects were studied: *Carausius morosus* (Sinety 1901), *Extatosoma tiaratum* (MacLeay 1827), *Peruphasma schultei* (Conle & Hennemann 2005), and *Sipyloidea sipylus* (Westwood 1859). The insects were reared at the Institute for General and Applied Zoology (*P. s*., *C. m*.) or at the Institute for Animal Physiology (*S. s*., *E. t*.), Justus-Liebig-Universität Gießen. *P. schultei* and *E. tiaratum* were kept as bisexually reproducing laboratory populations with male and female individuals. *C. morosus* and *S. sipylus* were reproducing by obligatory parthenogenesis and only females were used in experiments. Animals were kept in crowded laboratory cultures under a 12:12 light-dark regime (*S. s*., *E. t*.) or under long day conditions (*P. s*., *C. m*.), and were fed appropriate plant leaves.

Adults of all species were used for neuroanatomical studies, while we also investigated juvenile *P. schultei* (last larval instars) and 4^th^ larval instars of *S. sipylus*. Last larval instar (L6 for female, L5 for male; CvB, KS, YvB: unpublished observation) and adult animals of *P. schultei* were recognized by differences of the wing anlagen or wings, respectively. The postembryonic instars of *S. sipylus* were staged based on body size and morphology of wing anlagen according to Carlberg (1987) [74].

### Dissection for neuroanatomical investigation and tracing experiments

After cold anaesthesia of animals at 4 °C, the thorax and head were cut open on the dorsal side with scissors to gain access to the defence glands, the central nervous system and innervating nerves. To expose the SOG and its nerves, the cuticular tentorium covering it was cut medially with scissors. The animals were flattened out with insect pins in glass dishes filled with Sylgard (Sylgard 184, Suter Kunstoffe AG, Fraubrunnen, Switzerland) under *Carausius* saline [75, 76]; pH = 7.4). The oesophagus and gut were removed to expose the thoracic ganglia [75, 76]. The brain was exposed and during most preparations bisected with scissors in order to gain access to the suboesophageal ganglion and its nerves.

### Neuronal innervation of the defence glands

#### Nomenclature of nerves

The nomenclature of nerves from the suboesophageal ganglion and the thoracic ganglia follows the terminology established for *C. morosus* by Marquardt [34].

#### Analysis of nerves innervating the defence glands

Innervation patterns in the prothorax and head were studied by dissecting animals and staining of nerves in situ with the vital stain Janus Green B (0.02 %; [77]; dissolved in *Carausius* saline). The Janus Green B solution was stored at 4 °C, and cooled solution was applied to the tissues. The incubation lasted for 20–60 s, and the Janus Green B solution was immediately washed out repeatedly with *Carausius* saline. This incubation allowed to stain the nerves close to the defence glands.

Candidate nerves for gland innervation were further analysed by anterograde axonal tracing for endings on the gland. Different nerves were tested for a possible innervation of the defence glands in *P. schultei*, *C. morosus*, and *S. sipylus*: the *Nervus anterior* from the SOG, the *Nervus posterior* from the SOG, the *Nervus transversus*, and the *Nervus anterior* from the prothoracic ganglion (Fig. 4a; Table 1).

To investigate a gland innervation from neurons in the prothoracic ganglion via the *N. anterior* SOG, the connectives between suboesophageal ganglion and prothoracic ganglion were cut, and anterograde axonal tracing was employed on the cut connectives from the suboesophageal ganglion towards the gland in *S. sipylus* and *P. schultei* (Fig. 4b). Numbers of preparations for the tracing of the different nerves are summarised in Table 3.

**Table 3:** Numbers of gland preparations (*N*) using anterograde axonal tracing to study the gland innervation. In all tracing experiments, neurobiotin solution was used unless indicated otherwise

	*P. schultei*	*C. morosus*	*S. sipylus*
*N. anterior* SOG	19	4	12
T1-SOG connectives	8	(n. d.)	10
*N. posterior* SOG	20 (*n* = 8 traced with cobalt solution, *n* = 12 traced with neurobiotin solution)	4	15
*N. transversus*	18	8	20
*N. anterior* T1	6	8	20

*n. d* not determined

#### Identification of CNS neurons innervating the defence glands

Retrograde tracing of the nerves innervating the defence glands was used to reveal the neurons somata in the central nervous system. We traced the *N. anterior* SOG and smaller nerves at the defence gland in *P. schultei*, *S. sipylus* and *C. morosus*. Because the nerves of the intersegmental nerve complex (*N. posterior* SOG, *N. anterior* T1, and *N. transversus*) also innervate thoracic muscles close to the gland, only the terminal nerve branches contacting the gland were traced in retrograde fills (Fig. 4c). The nerve branches were cut close to the gland for tracing, and the SOG and T1 ganglion were excised after incubation. In some preparations in *P. schultei*, the brain was kept intact as the nerve from the suboesophageal ganglion (*Nervus anterior* SOG) was traced from the defence glands in retrograde direction towards the central nervous system (CNS) (Table 2). In *P. schultei* and *S. sipylus*, we also studied the T2 and T3 ganglia for the presence of stained neurons. In *E. tiaratum*, only the *Nervus anterior* from the SOG was studied.

### Axonal tracing methods

Axonal tracing experiments were carried out *in situ* after the dissection as described above. For both anterograde and retrograde directions of tracing we used either neurobiotin (5% in *Aqua dest*.; Vector Laboratories, Burlingame, CA.) or cobalt chloride (5% in *Aqua dest*.; Merck, Darmstadt, Germany), for details see below. For Orthoptera, Honegger et al. [52] noted that neck muscle motoneurons did not stain with lower concentrations of cobalt than 5% [52]. The results obtained here with both methods were consistent on both glands and ganglia if used in parallel (see Tables 2, 3). Neurobiotin solution was used for most retrograde tracing experiments, as in ganglia the overall contrast of staining and the quality of labelling neurite branches was better with this tracer. Neurobiotin was used mainly for anterograde tracing of nerves innervating the defence glands, since the incubation of glands in ammonium solution for chloride precipitation (detailed below) occasionally resulted in a dark staining of the gland surface.

For axonal tracing, the nerves were cut midway between the respective ganglion and the defence glands with iridectomy scissors (Fig. 4). The free end of the nerve was then transferred into a glass capillary filled with tracer solution. The preparations were incubated for 4 days at 4 °C.

For tracing with cobalt chloride [78, 79], the tracer was precipitated by incubation in a 1 % ammonium sulfide (Fluka, Buchs, Switzerland) solution in *Carausius* saline for 15 min [80]. The glands and ganglia were fixed in 4 % paraformaldehyde (Sigma Chemicals, St. Louis, Missouri) in phosphate buffer (0.04 mol/l Na_2_HPO_4_, 0.00574 mol/l NaH_2_PO_4_ x 2 H_2_O; pH = 7.4) for 60 mins. They were then briefly rinsed in phosphate buffer. Preparations were not silver-intensified.

Neurobiotin as a tracer was revealed in the nerve fibres by processing glands with the Avidin-Biotin-kit (Vectastain ABC Kit PK-6100; Vector Laboratories, Burlingame, CA) and DAB solution (Vectastain DAB Kit SK-4100; Vector Laboratories) according to manufacturer’s instructions. Glands were either mounted and viewed in phosphate buffer, or dehydrated via a graded ethanol series (Carl Roth, Karlsruhe, Germany) at 10 min incubation for each step and finally cleared in methyl salicylate (Fluka).

Ganglia were fixed following tracing with neurobiotin solution in 4% paraformaldehyde dissolved in phosphate buffer for 60 min. They were stored in PBST buffer (0.1369 mol/l NaCl, 0.0027 mol/l KCl, 0.01 mol/l Na_2_HPO_4_, 0.00176 mol/l KH_2_PO_4_, [all from Merck, Darmstadt, Germany], 0.1% Triton X-100 [Roth, Karlsruhe, Germany]; pH = 7.2). They were then dehydrated via a graded ethanol series, incubated in xylene (Fluka) for 5 min, and rehydrated via a graded ethanol series. Afterwards, they were incubated in a solution of collagenase and hyaluronidase (Sigma Chemicals; 1 mg in 1 ml phosphate buffer of each enzyme) in phosphate buffer at 37 °C for 60 min and washed repeatedly in phosphate buffer. As for glands, intracellular neurobiotin in ganglia was visualised with the Avidin-Biotin-kit (Vectastain ABC Kit PK-6100; Vector Laboratories, Burlingame, CA) and DAB solution (Vectastain DAB Kit SK-4100; Vector Laboratories) according to manufacturer’s instructions. Ganglia were dehydrated in a graded ethanol series and cleared in methyl salicylate.

As a control against false positive staining of potential biotin-rich neurons [81], we dissected ganglia from *P. schultei* and *S. sipylus* (five SOG and prothoracic ganglia from each species) without previous tracing with neurobiotin and processed them as described above for fixation and ABC staining. In nerve tracing control experiments the CNS neurons were not stained. However, in *S. sipylus*, few somata in the prothoracic ganglion were stained rather weakly, while in *P. schultei*, one bilaterally occurring neuron soma was occasionally stained in the anterior suboesophageal ganglion and somata of 3–8 neurons were stained in the prothoracic ganglion in a ventral posterior position at the midline or slightly laterally. At these positions within the ganglia, none of the neurons detectable by the filling experiments are present. In particular, the neurons stained by tracing of the intersegmental nerve complex (*N. anterior* of the prothoracic ganglion) are located in the dorsal ganglion. Further, for false-positive stains, the nuclei were described to be excluded from staining [81], while in this study the neurons somata in the ganglia detected by retrograde tracing were densely stained throughout (Figs. **7**, 10). The controls thus support the genuine staining of CNS neurons by retrograde axonal tracing with neurobiotin.

### Documentation

Innervation patterns revealed with Janus Green B staining in situ were documented by drawing with a Leica drawing mirror attached to a Leica dissection microscope.

Isolated glands and ganglia were viewed in phosphate buffer or in methyl salicylate on an Olympus BH-2 microscope and photographed with a Leica DCF-320 camera [2088 x 1055 pixel] attached to the microscope. Some preparations were photographed in series and stacked pictures were generated using the freeware program CombineZP (http://www.hadleyweb.pwp.blueyonder.co.uk/).

Ganglia were drawn with help of a drawing attachment on a Leitz Dialux microscope (Leitz, Wetzlar, Germany) and redrawn in ink.

Photomicrographs were carefully adjusted for overall brightness and contrast using Corel PhotoPaint (Corel, Ottawa, Canada). Figure panels were assembled and labelled using CorelDraw 11 (Corel, Ottawa, Canada).

### Electrical stimulation of nerves contacting the defence glands in *P. schultei*

To test the nerves physiologically, animals were dissected as described above prior to tracing experiments under a dissection miscroscope (Hund, Wetzlar, Germany). The nerves were individually stimulated by a double silver hook electrode. Stimuli were generated by a Grass SD 5 Stimulator (Grass Medical Instruments, Quincy, Massachussets, USA). Stimuli used were of variable duration and amplitude, but responses in form of gland contractions could be elicited by stimuli from 0.3 V. We tested nerves of the SOG, the SOG-T1 connective or the intersegmental nerve complex independently by cutting the other nerves with iridectomy scissors.

## Additional file

Additional file 1:
**Homologous nerves and neurons innervating defence glands in stick insects or neck muscles in related insects.** (DOCX 13 kb)

## Abbreviations

CCAP: Crustacean cardioactive peptide
CLN: Contralateral neurons
DN: Dorsal neuron
DUM neuron: Dorsal unpaired median neuron
ILN: Ipsilateral neuron
ISN: Intersegmental nerve complex
PIN: Prothoracic intersegmental neuron
SMN: Suboesophageal midline neuron
SN1: Salivary neurone 1
SN2: Salivary neurone 2
SOG: Suboesophageal ganglion
T1: Prothorax
T2: Mesothorax
T3: Metathorax
VMN: Ventral medial neurons
