# Supplementary material for: Neurons of self-defence: neuronal innervation of the exocrine defence glands in stick insects
Source: Front Zool. 2015 Oct 24;12:29. doi: 10.1186/s12983-015-0122-0 (PMC4619533; doi:10.1186/s12983-015-0122-0)
Supplement: Additional file 1: — Homologous nerves and neurons innervating defence glands in stick insects or neck muscles in related insects. (DOCX 13 kb) [file 12983_2015_122_MOESM1_ESM.docx]

**Supplementary table 1: Homologous nerves and neurons innervating defence glands in stick insects or neck muscles in related insects**

(-) indicates no homologous neuron or neurons have been identified.

*Taxon*  **Phasmatodea Locust Cricket Blattodea**

*Species Peruphasma schultei Locusta migratoria Gryllus campestris Periplaneta americana*

*Carausius morosus Schistocerca gregaria*

*Sipyloidea sipylus*

*Reference* present study Altman and Kien 1979 Honegger et al. 1984 Davis 1983

Honegger et al. 1984

**SOG nerve** *N. anterior* SOG Nerve 6 Nerve 6 Tergal nerve

**SOG neurons** 2-4 VMN 2 – 3 motoneurons of 3 – 4 motoneurons of 2 DL_1_

**via nerve 6** muscle 50/ 51 muscle 50/ 51

1 ILN - - -

1 CLN - - 1 VCL?

**via *N. posterior* SOG** 1 ventral SMN via - 1 SOG neuron via N8 DL_3_

intersegmental nerve complex ISN

**T1 neurons**

**via *N. anterior* SOG** 1 PIN - - -

**via *N. anterior* T1** 1-2 DUM via ISN DUM ? (only axon reported) DUM

1 anterior DN - - -

Abbreviations:

CLN contralateral neuron

DL_1/ 3_ dorsal longitudinal motor neurons _1/ 3_

DN dorsal neuron

DUM dorsal unpaired median neuron

ILN ipsilateral neruon

ISN intersegmental nerve complex

PIN prothoracic intersegmental neuron

SMN subesophageal midline neuron

SOG subesophageal ganglion

T1 prothoracic ganglion

VCL ventral contralateral neuron

References:

Altman JS, Kien J (1979) Suboesophageal neurons involved in head movements and feeding in locusts. Proc R Soc Lond B 205: 209 – 227

Davis NT (1983) Serial homologies of the motor neurons of the dorsal intersegmental muscles of the cockroach, *Periplaneta americana* (L.). J Morphol 176: 197

– 210

Honegger H-W, Altman JS, Kien J, Müller-Tautz R, Pollerberg E (1984) A comparative study of neck muscle motor neurons in a cricket and a locust. J Comp

Neurol 230: 517 – 535
